# A policy analysis of policies and strategic plans on Maternal, Newborn and Child Health in Ethiopia

**DOI:** 10.1186/s12939-022-01656-x

**Published:** 2022-05-19

**Authors:** Josea Rono, Lynette Kamau, Jane Mwangwana, Jacinta Waruguru, Pauline Aluoch, Maureen Njoroge

**Affiliations:** 1 E&K Consulting Firm, Nairobi, Kenya; 2 African Population and Health Research Center, Nairobi, Kenya

**Keywords:** Health policy, Health systems, Health systems research, Sustainable development goals, Maternal, Newborn and child health, EquiFrame, Ethiopia

## Abstract

Significant progress has been made to advance Maternal, Newborn and Child Health (MNCH) in Ethiopia. Further, the country has enshrined equity as a core value in their strategic and development frameworks and policies. Although national statistics show improved health outcomes, there exists persistent inequities in avoidable health risks and premature deaths. Additionally, the improving health statistics mask the disparities in health outcomes based on education, employment status, income level, gender and ethnicity dimensions.

The EquiFrame framework was used to assess the extent to which equity was entrenched in MNCH health policies and plans. The framework, which describes core concepts against which health policies and plans can be assessed, also provides a scoring criterion for policy assessment. The framework was modified to include the concept of intersectionality, which is increasingly gaining significance in the health policy ecosystems. The policies and plans reviewed in this analysis exercise were selected based on (1) their relevance – only policies and plans in force as of the year 2020 were considered; (2) availability in the public domain as this study was limited to desk research; and (3) relevance to MNCH. A total of five policies and plans were analyzed and evaluated against the 15 core concepts presented in the modified EquiFrame framework. Following the outcomes of the assessment, documents were ranked as either being low, moderate, or high, in exhaustively addressing the core concepts.

The Ethiopia Health Sector Transformation Plan (2016–2020) is the only policy or plan that earned a high ranking. The other four policies and plans were ranked as moderate. This shows that while majority of the Ethiopian health sector policies and plans exist and address the core health equity concepts, they fail to: (i) spell out plans to implement and monitor the proposed interventions; and (ii) demonstrate evidence that the interventions were implemented or monitored. With the global goal of leaving no one behind, future policy development in Ethiopia needs to prioritize equity considerations in order to enhance the ongoing health improvement.

## Background

Whitehead defines equity as follows in a widely regarded 1992 paper on the concepts and principles of equity in health: “the absence of avoidable, unfair, or remediable differences among groups of people, whether those groups are defined socially, economically, demographically or geographically or by other means of stratification.” [1].

The Sustainable Development Goals (SDGs) aspire to adopt this broader and sustainable approach to address inequities in health [2]. The SDGs, unlike the Millennium Development Goals (MDGs) before them, not only represent a potentially transformative development agenda but are also more explicit in their strong focus on inclusion and reduction of inequalities [2]. Specific equity components on MNCH are captured in SDG 3.1 which aims to accelerate momentum on ending preventable maternal, newborn, and child deaths, and SDG 3.7 which aims to ensure universal access to sexual and reproductive health-care services [3].

With the endeavors to achieve the global goals, significant health gains as evidenced by improvements in health indicators such life expectancy at birth and infant and child survival have been made [4]. Despite this progress, health inequity remains persistent in the health system [5]. Failure to achieve health equity may be attributed to factors such as complexity in decision-making in health system contexts and absence of mechanisms that allow for communities to participate in, claim and benefit from resources and entitlements in national and regional health systems [6, 7]. The allocation of resources for health in Africa is sometimes driven by deep-rooted political and economic interests, which propagate inequities [6].

### Equity / inequity in MNCH

The Ethiopian Government was guided by its pro - poor policies and strategies and invested heavily in health system strengthening (HSS) [8]. Specifically, HSS is guided by the WHO six building blocks; with implementation steered by the Ethiopia Ministry of Health with support from multiple stakeholders, including the ministry of finance; development partners; Civil Society Organizations; and the community. Continuing evidence shows that government consideration for community input when designing policy is likely to lead to improved health outcomes [9].

Interventions under the HSS programme include the country’s flagship program, called the Health Extension Programme (HEP). HEP delivers cost effective basic services to all Ethiopians, mainly women and children. HEP is underpinned by the core principle of community ownership that empowers communities to manage health problems specific to their communities, thus enabling them to produce their own health. Through the programme, more than 38,000 Health Extension Workers (HEWs) have been trained and deployed all over the country, availing two HEWs in every Kebele (a cluster of villages) [8]. The Kebele System is a tight system of neighborhood administration and control in Ethiopia, with every Kebele office comprising around 500 households per unit [10].

The HSS focus has resulted in significant gains to health status of Ethiopians. For instance, under-five mortality rates per 1000 live births dropped from 140 in the year 2010, to 51 in the year 2019 [11]. Similarly, neonatal morality rate dropped from 38 in the year 2010 to 28 in the year 2019 [11]. Between the years 2000 and 2017, maternal mortality rates per 100,000 live births reduced from 1030 to 401 [12]. Despite this progress, Ethiopia remains to be one of the six countries who collectively account for more than half the maternal deaths in the world [13].

While there is significant MNCH progress at national level, inequities exist across different geographic regions, urban and rural areas and socioeconomic groups [14]. Health inequality is driven by low economic status, illiteracy, rural residence, no occupation and lack of access to mass media [15]. For instance, more urban women are likely to receive Antenatal care (ANC) from a skilled provider compared to rural women (85% and 70%, respectively). Additionally, more urban women are likely to have four or more ANC visits compared to rural women (59% and 37%, respectively) [16]. Further, 79% of women with a secondary education received four or more ANC visits, compared to 32% of women with no education. Receiving ANC from a skilled provider is important to monitor pregnancy and has also proven to reduce morbidity and mortality risks for the mother and child during pregnancy, delivery, and the postnatal period [16].

Global evidence suggests that MNCH inequities are partly related to intersectional gender-related disparities [17]. These disparities are caused by policies failing to discuss multi-stakeholder dialogues with female representatives from minority groups; and patriarchal norms which inhibit women from accessing health services [17, 18].

### Stakeholders within MNCH policy landscape

Over the past decade, Ethiopia has enjoyed unprecedented support from its development partners. The support has been guided by national aspirations and plans including: The Constitution and Ethiopia Health Sector Transformation Plan (HSTP) [8]. Ethiopia MNCH programmes take on a multi-stakeholder approach with key stakeholders involved including: the Ministry of Health, regional Governments, donors, development partners, civil society organizations, training institutions, and the community. The one plan-one budget and one report is the approach used by the Ministry of Health to coordinate efforts of stakeholders within the health sector, with the approach ensuring alignment and harmonization in stakeholder work [19]. However, stakeholder engagement faces challenges around inclusive stakeholder engagement in MNCH policy and planning, as well as transparency in information sharing [9, 20].

### The EquiFrame analysis framework

In order to anchor the equity analysis on empirical evidence, it was imperative that an analysis framework be identified and/or developed. Examples of frameworks that have been fronted to guide the assessment and improvement of equity in health policy development and implementation include: Health Equity Impact Assessment (HEIA); Health Equity Assessment Tool (HEAT); and EquiFrame framework [21–23]. While the plethora of frameworks is wide, the EquiFrame framework [23] was found to be the most pragmatic starting point for the purposes of this retrospective analysis of already developed policies and plans. The framework describes core concepts against which health policies and plans can be assessed and also provides a scoring criterion to guide the assessment. The application of the framework exposes significant inequalities between policies and plans in terms of vulnerable groups coverage and human rights core concepts covering [24]. The application of the framework has been previously used to assess sexual and reproductive health polices in Ukraine, Scotland, Moldova, and Spain, with the Spanish policy performing best and the Ukrainian policy the worst [24]. The EquiFrame has also been used to compare 51 health and welfare policies from Namibia, Malawi, South Africa, and Sudan, showing that marginalized and vulnerable people may experience greater social exclusion in low-income countries compared to wealthier countries [25].

Within the EquiFrame framework, core concepts may be added or removed to suit specific requirements, political, cultural or other contextual interests or constraints [24]. While the core concepts presented are quite comprehensive, they do not address some of the more recently developed aspects of equity assessment. For this reason, the EquiFrame framework was modified by the incorporation of a critical albeit a more recently developed concept of intersectionality [26], that is increasingly gaining significance in the health policy ecosystem [27, 28]. When a reference to a core concept was identified, the extent to which the concept was addressed was ascertained using the definition of the concepts extracted from literature [23].

**Fig. 1:**
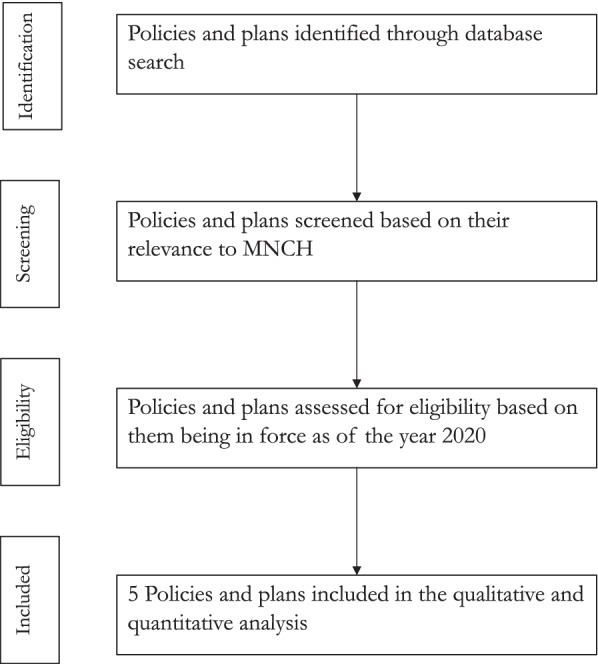
PRISMA flow diagram

Taken together, the key concepts adopted for this analysis are listed in Table 1. Figure 1 describes the step-by-step methodology taken to identify plans and policies for review.

**Table 1:** Key definitions of core concepts

Core concept	Definition
**Participation/ inclusivity in development of policies and plans**	This looked at the extent to which a policy or plan provided for (i) mechanisms (or representation) for communities to participate in, claim and benefit from entitlements in national systems and/or (ii) stakeholder engagement in its development and/or (iii) dissemination to key stakeholders or the general public
**Equity considerations in budgets and resource allocation**	This looked at the extent to which policy or plan incorporated inequity metrics (e.g. deprivation) in public budget resource allocation
**Non – discrimination**	This examined whether the policy or plan supported the rights of vulnerable groups to have equal opportunity in receiving health care
**Individualized services** (also referred elsewhere as acceptability [26])	This examined the extent to which a policy or plan supported the rights of vulnerable groups to receive individually tailored services to meet their needs and choices
**Entitlement**:	This looked at whether the policy or plan stipulated how vulnerable groups qualified for specific benefits relevant to them
**Access**	This looked at whether the policy or plan supported vulnerable groups in terms of their physical, economic, and information access to health services
**Quality**	This examined whether the policy or plan supported quality services to vulnerable groups through highlighting the need for evidence-based and professionally skilled practice
**Efficiency**	This examined the extent to which a policy or plan supported efficiency by providing a structured way of matching health system resources with service demands to address health needs of vulnerable groups
**Effectiveness** (Evidence-based focus)	This examined whether the policy or plan used best evidence and practises to optimize health outcomes.
**Accountability**:	This examined whether the policy or plan under review specified to whom, and for what, services providers were accountable
**Do no harm/ Safety**	This examined whether the policy or plan under review provided for minimization of risks during healthcare delivery
**Cultural responsiveness**	This looked at whether the policy or plan under review ensured services responded to the beliefs, values, gender, interpersonal styles, attitudes, cultural, ethnic, or linguistic, aspects of the person.
**Intersectionality**	This examined whether the policy under review took into account intersectionality i.e. going beyond the singular categories that are typically favoured in equity-driven analyses (e.g., sex and gender in sex and gender based analysis) and also beyond the kind of enumerated list of determinants of health often found in health impact assessments (e.g. residence, wealth etc.) to consider interactions between categories and determinants simultaneously e.g. impact of gender and education level simultaneously [26].
**Human resources for health (HRH)**	This looked at whether the policy or plan provided for the development (e.g. through training) and maintenance of appropriate HRH to ensure adequate provision of quality healthcare services to women and children
**Infrastructure**	This examined whether the policy or plan provided for the development and maintenance of appropriate infrastructure to ensure adequate provision of quality healthcare services to women and children

## Methods

The analysis and scoring approach provided by the EquiFrame framework was found to be simple and pragmatic and was adopted in scoring the different policy and planning documents reviewed as part of this analysis. The Framework was used to assess five key health policies and plans on MNCH in Ethiopia. The goal was to determine the extent to which existing health policies and plans supported mother and child health-related human rights and identify policies that needed to be revised. The policies and plans that were chosen for examination were the most recent in the public domain that were relevant to MNCH. The documents analyzed were:

1. Ethiopia Health Sector Transformation Plan (2016–2020) [8]
2. National Health Sector Strategic Plan for Early Childhood Development in Ethiopia (2020/21–2024/25) [29]
3. National Adolescent and Youth Reproductive Health Strategy (2016–2020) [30]
4. Ethiopia National Health Care Quality Strategy (2016–2020) [31]
5. National MNH Quality of care roadmap (2017/18–2019/20) [32]

The analysis of the policies and plans was assessed according to the scale proposed by the framework in Table 2. Each document was given an overall summary ranking in terms of it being of low, moderate, or high standing according to the criteria provided in Table 3. The rating was based on the quality of commitment to each individual core concept within the document.

**Table 2:** Core concept scoring table

Scoring	Description of scoring category
**0**	Concept not mentioned
**1**	Concept only mentioned
**2**	Concept mentioned and explained
**3**	Specific policy actions identified to address the concept
**4**	Intention to monitor the concept was expressed
**5**	Evidence that the specific policy action is (or was) monitored

**Table 3:** Equity analysis of MNCH policies and plans tool

Analysis	
Broad Analysis Category	Description of Analysis Category
**Core concept coverage**	The percentage of core concepts (CCs) included in the policy out of the total number of CCs. CC coverage = (*n*/15) × 100 (where: n is the number of core concepts that score above 0; and 15 is the total number of CCs).
**Core concept quality**	The percentage of CCs rated as four or five (based on Table 1 scoring), that is, as either stating intention to monitor a specific policy action or there is evidence that the specific policy action is (or has been) monitored. CC quality = (Total number of CCs scoring four or five /15) × 100 (where: 15 is the total number of CCs)
**Overall ranking of policy or plan document**	Policies and plans were ranked based on their performance scores in the CC coverage and quality categories. The ranking was given as below: (i) High = if the policy achieved ≥50% on both scores above. (ii) Moderate = if the policy achieved ≥50% on one of two scores above. (iii) Low = if the policy achieved < 50% on both scores above.

### Quality assurance

The policies and plans were presented in a stakeholder validation meeting, with key participants including community representatives, Ministry of Health, and Civil Society Organizations. Through the validation meeting, stakeholders were able to look objectively at the analysis and recommend changes to enhance the analysis.

## Results

Based on the modified EquiFrame analysis framework, four of the five health policies and plans observed in this study ranked as moderate. The only exception is the Ethiopia Health Sector Transformation Plan (2016–2020) which ranked as high. None of the policies considered ranked low, with 80% of equity concepts mentioned across all policies and plans (Table 4). The scoring (Table 5) was arrived at through rating the quality of commitment to the individual core concepts within the policies and plans. However, the policies and plans did not: (i) spell out plans to implement and monitor the proposed interventions; and (ii) demonstrate evidence that the interventions were implemented or monitored.

**Table 4:** Results of the equity analysis of MNCH policies and plans

	**Strategy**				
**Core concept**	**Ethiopia Health Sector Transformation Plan**	**National Health Sector Strategic Plan for Early Childhood Development in Ethiopia**	**National Adolescent and Youth Reproductive Health Strategy**	**Ethiopia National Health Care Quality Strategy**	**Ethiopia National MNH Quality of Care Roadmap**
Participation/ inclusivity in development of policies and plans	4	3	4	3	3
Equity considerations in budgets and resource allocation	4	4	4	4	1
Non - discrimination	3	3	3	3	3
Individualized services	3	3	3	4	2
Entitlement	3	2	3	1	1
Access	4	4	3	4	4
Quality	4	4	4	4	4
Efficiency	4	3	4	2	1
Accountability	3	2	3	3	4
Do no harm/ Safety	3	0	3	4	3
Cultural responsiveness	3	1	1	0	0
Effectiveness (Evidence-based focus)	4	4	4	4	4
Intersectionality	0	0	0	0	0
Human resources for health	4	4	3	3	3
Infrastructure	4	3	3	3	3

**Table 5:** Ranking of the equity analysis of MNCH policies and plans

	**Strategy**				
**Core concept**	**Ethiopia Health Sector Transformation Plan**	**National Health Sector Strategic Plan for Early Childhood Development in Ethiopia**	**National Adolescent and Youth Reproductive Health Strategy**	**Ethiopia National Health Care Quality Strategy**	**Ethiopia National MNH Quality of Care Roadmap**
Core concept coverage	93%	87%	93%	87%	87%
Core concept quality	53%	33%	33%	40%	27%
Overall ranking of policy or plan document	High	Moderate	Moderate	Moderate	Moderate

Regarding quality of commitment to core concepts, the concepts of quality and effectiveness received the highest score of 4. This shows an intention to monitor these concepts was expressed. In terms of specific concepts, most of the policies and plans failed to mention two important concepts namely: cultural responsiveness, and intersectionality. Intersectionality was not mentioned in any of the policies and plans considered. Ideally, intersectionality can enable peoples subjugated in different but connected ways to unite around more expansive agendas for social justice. Cultural responsiveness can be addressed through leveraging community networks such as community health workers, religious organizations, traditional leaders, and educational institutions to address the disparities [9]. Community engagement has proved a useful tool in ensuring policies are responsive to the needs of the community and that people in underprivileged communities have a better understanding of how to manage various health issues [9].

In addition, the policies and plans do not put forward any monitoring and evaluation (M&E) frameworks or indicators that are pro-equity and all indicators outlined in the document track macro (population-wide) metrics.

## Discussion

This review demonstrated that while there is remarkable progress in MNCH outcome, these improvements mask the inequities experienced across unique population groups such a women and children, with gaps based on different social factors, including but not limited to education, employment status, income level, gender and ethnicity. Despite these equity deficits, MNCH policies and plans in the country score moderately in terms of their equity-related provisions. Most of the policies and plans fail to adopt equity consideration in the entire development and implementation lifecycle. A life cycle approach in policy is likely to enable better uptake of MNCH services [17]. These insights are particularly important, given the need to adjust current provisions of Universal Health Coverage in Africa in line with the population’s needs and realities.

Given both the broader focus on the health sector and concerns about health equity, donors have offered significant financial and technical support to MNCH in Ethiopia [14]. While there has been improvement in the sector, financial and regulatory constraints are perceived as limiting the country’s ability to provide more equitable health services. Some of the priority areas in Ethiopia that need urgent attention include: a sustained and strong political will to develop the health sector; an increase in financial and human resources to support the health sector; and better government accountability to its citizens [14, 33].

Establishing goals and targets that aim to reduce inequity encourages the orientation of policies, programmes and practices to promote health in disadvantaged population groups. Without a dedicated focus on equity within monitoring and evaluation frameworks in policies, efforts to improve MNCH will be slowed down.

In light of best practice and the equity analysis results presented above, this review makes the following recommendations:

### The government should adopt intersectionality in equity measurement

Despite intersectionality being a significant aspect in the health policy ecosystem, the MNCH policies and plans examined here do not include it as a concept. Future measurement of health outcomes should move beyond tracking inequity along individual dimensions/ categories (e.g. residence and gender) and incorporate intersectionality. Among the ways of incorporating intersectionality is through promoting social science approaches and the use of such evidence for policy making to capture the nuance and complexity of intersecting factors. This will allow for the identification of sub-populations at the intersection of multiple dimensions that merit tailored policy interventions that may not be evident at the macro (population-wide) level [34].

### The Ministry of Health needs to incorporate a more robust M&E framework to track progress and inform decision making for MNCH programmes

The moderate scores earned by existing MNCH policies and plans allude to sub-optimal integration of M&E frameworks. Future MNCH policy development ought to not only describe and analyze the distribution and causes of inequalities in health, but also include measurable indicators to track progress on reducing inequities. M&E can help improve governance in a variety of ways. First, the data gathered can be used in government decision-making and prioritization of activities, particularly during the budgeting process. Second, it can assist policy makers by giving information (especially on costs and benefits) that can be used in planning or monitoring the progress of ongoing initiatives [35].

## Limitations

The issues in the public sector are complex, with limits existing not only in the policy analysis process, but also in the application of analysis. Policy alternatives that sound great on paper may not hold up in practice. This limitation can be solved by acknowledging that there is unlikely to be a one-size-fits-all model that would result in easy, actionable recommendations.

## Conclusion

The findings presented here indicate that future efforts should go beyond improving MNCH outcomes at the macro level and more into enhancing the adoption of equity considerations in the entire MNCH policy development and implementation lifecycle, including M&E. These insights imply that future policy development in Ethiopia ought to deliberately take into account equity consideration in order to enhance equitable improvement in health outcomes and live up to the SDGs central promise of leaving no one behind.

## Data Availability

The datasets used and/or analyzed in the review/analysis are available from the corresponding author on reasonable request.

## Abbreviations

APHRC: African Population and Health Research Center
AYRH: Adolescent and Youth Reproductive Health
CCs: Core concepts
ECD: Early Childhood Development
HEP: Health Extension Programme
HEWs: Health Extension Workers
HSTP: Ethiopia Health Sector Transformation Plan
MNCH: Maternal, Newborn and Child Health
MDGs: Millennium Development Goals
M&E: Monitoring and Evaluation
SDGs: Sustainable Development Goals
WHO: World Health Organization
