# Correction: A policy analysis of policies and strategic plans on Maternal, Newborn and Child Health in Ethiopia

**DOI:** 10.1186/s12939-022-01680-x

**Published:** 2022-06-03

**Authors:** Josea Rono, Lynette Kamau, Jane Mangwana, Jacinta Waruguru, Pauline Aluoch, Maureen Njoroge

**Affiliations:** 1 E&K Consulting Firm, Nairobi, Kenya; 2 African Population and Health Research Center, Nairobi, Kenya

**Correction: Int J Equity Health 21, 73 (2022)**

**https://doi.org/10.1186/s12939-022-01656-x**

After publication of this article [1], the authors reported that the author name Mangwana was incorrectly written as Mwangwana.

The original article [1] has been updated.
